# Periprocedural myocardial and renal injury in patients undergoing elective percutaneous coronary interventions – is there an association?

**DOI:** 10.1097/MD.0000000000016989

**Published:** 2019-10-25

**Authors:** Mario Stipinovic, Luka Percin, Vedran Radonic, Helena Jerkic, Ivana Jurin, Tomislav Letilovic

**Affiliations:** a Division of Cardiology, Department of Medicine, University Hospital Merkur, Zagreb; b Department of Emergency Medicine of the Primorje-Gorski Kotar Country, Rijeka; d School of Medicine, University of Osijek, Osijek; c Division of Cardiology, Department of Medicine, University Hospital Dubrava, Zagreb; e School of Medicine, University of Zagreb, Zagreb, Croatia.

**Keywords:** contrast-induced nephropathy, early creatinine shift, major adverse cardiovascular events, periprocedural myocardial injury

## Abstract

Periprocedural myocardial injury (PMI) and contrast-induced nephropathy (CIN) are frequent complications of percutaneous coronary intervention (PCI) associated with early and late major adverse cardiovascular events. Both conditions are associated with similar risk factors, which could imply their possible association. The aim of our study was to assess the correlation of PMI and early postprocedural creatinine shift (ECS) as a marker of renal injury.

A total of 209 hospitalized patients with stable coronary artery disease (CAD) were enrolled, who underwent an elective PCI in a period of 12 months. All patients had their serum high-sensitivity troponin I (hsTnI) measured at baseline and 16 hours after the PCI. PMI was defined according to the elevation of postprocedural hsTnI using criteria provided by both the most recent consensus documents as well as evidence-based data. Renal injury was evaluated using the ECS concept. Serum creatinine (SCr) was also measured at baseline and at 16 hours. ECS was defined as SCr >5% at 16 hours compared to baseline.

Although incidence of both PMI (77.5%) and ECS (44.5%) were high, no association of these 2 conditions could be found. Further analyses of our data showed that diabetes is associated with a higher incidence of ECS, while patients on beta-blocker therapy had a lower incidence of ECS.

In our study, no association between PMI and ECS was found. Additional studies with a larger number of patients and longer patient observation are needed to assess the correlation between PMI and CIN as well as to validate the attractive, but controversial, concept of ECS as an early marker of CIN.

## 1 Introduction

Percutaneous coronary intervention (PCI) is considered as the primary method of coronary revascularization in patients with single or double vessel coronary artery disease (CAD).^[1]^ The high prevalence of CAD, together with the proven efficacy of PCI in certain clinical settings, has resulted in widespread use of this procedure. Unfortunately, PCI is also related to certain adverse events. Periprocedural myocardial injury (PMI) and contrast-induced nephropathy (CIN) are relatively frequent and extensively studied unwanted consequences of PCI. As such, they represent a significant health problem.^[2,3]^

PMI occurs in 5% to 30% of PCI. It is associated with significantly increased risk of major adverse cardiovascular events.^[4]^ It can present as a periprocedural infarction with clinical, electrocardiographic, and/or echocardiographic features of myocardial damage. On the other hand, PMI can be clinically silent and detected only through the postprocedural rise of troponin.^[5–7]^ The risk factors for the development of PMI are many and can be divided into patient-related, lesion-related, and procedure-related factors.^[7]^

CIN is another important complication of PCI. It represents acute kidney injury as a result of an intravascular iodinated contrast application in the absence of an alternative cause.^[8]^ CIN incidence after PCI varies between less than 1% to more than 20% and even up to more than 50% in some high-risk subgroups. CIN is associated with greater morbidity, mortality, and longer hospital stays.^[3,9,10]^ It is detected as a rise of serum creatinine (SCr), 48 to 72 hours after contrast administration. In the modern era of the same-day discharge (SDD) PCI approach, several author groups have searched earlier markers of renal damage. Early creatinine shifts (ECSs), as early as 12 hours after PCI, have been shown to be significant predictors of CIN and persistent renal damage.^[11,12]^ As with PMI, risk factors can be divided into patient-related^[13,14]^ and procedure-related factors.^[15]^

Considering the significant overlap in both patient-related and procedure-related risk factors of PMI and renal injury, one would expect a significant association of these conditions, and if proven so, PMI may have the potential to be an early marker for CIN in clinical practice. However, data on such an association does not exist. The aim of this study was to investigate the possible association of PMI and renal damage detected as ECS.

## 2 Methods

This cross-sectional study included 209 consecutively hospitalized patients with a stable CAD who underwent an elective PCI at Merkur University Hospital, Zagreb, Croatia, between December 2016 and December 2017. The study protocol was reviewed and approved by the Merkur Hospital ethics committee and all patients provided their informed consent.

All patients had stable coronary disease with documented inducible myocardial ischemia. Stable patients were defined as those with no recent deterioration of pain in the previous 2 months or without rest angina in the previous 48 hours. For each patient, we calculated the glomerular filtration rate using the Modification of Diet in Renal Disease (MDRD) study's 4 variable equation: estimated glomerular filtration rate (eGFR) = 186.3 × (SCr mg/dL)^−1.154^ × age^−0.203^ × (0.742 if female) × (1.21 if black).^[16]^ Only patients with eGFR = 40 mL/minute/1.73 m^2^ were included in the study. Further criteria for inclusion were that the PCI procedure was successful and that the optimal final result was obtained (ie, a thrombolysis in myocardial infarction flow grade 3 in the treated vessel with a residual stenosis less than 20% by quantitative coronary angiography [CA]).

The exclusion criteria were: age less than 18, acute coronary syndrome, acute kidney injury, baseline glomerular filtration less than 40 mL/minute/1.73 m^2^, major (more than 1.5 mm) side branch occlusion, major hemorrhage within 4 weeks or contraindication to the use dual antiplatelet therapy, unsuccessful procedures, or target lesion in saphenous graft.

Immediately before PCI procedures, a bolus of unfractionated heparin was administered according to a standardized protocol. PCI procedures were performed using 2 types of low osmolar contrast media (CM): iodixanol or ioversol.

Blood samples were collected before PCI and at 16 hours after PCI. We assessed the samples’ values of serum high-sensitivity troponin I (hsTnI) and SCr. The hsTnI and SCr values were measured using the standardized protocol of the institutional laboratory. The upper reference limit (URL) of serum hsTnI was 34.2 ng/L for men and 15.6 ng/L for women. PMI was defined using 2 different approaches. Firstly, we used the most recent definition provided by the European Society of Cardiology (ESC) was used. According to the ESC consensus document (which refers to standard and not high-sensitivity troponin [hsTn]), PMI is diagnosed as a postprocedural increase in TnI. Using the cut-offs provided by that definition, PMI was divided into PMI of low degree (hsTnI increase <5x URL) and PMI of high degree (hsTnI increase ≥5x URL) if basal hsTnI was <URL. If basal hsTnI was >URL, then an increase of >20% of the basal value was considered to be a PMI of high degree, and an increase of <20% of the basal value was considered to be a PMI of low degree.^[6]^ The second definition that was used was extrapolated from the work of Koskinas et al and Zeitouni et al^[17,18]^ They investigated PMI with hsTn measurements. Using their approach, we defined PMI as an increase of hsTnI ≥7x URL or an incremental increase of hsTn ≥7x URL in those with elevated baseline values. As both study groups showed these cut-offs to be related to major adverse cardiovascular events, we considered PMI diagnosed using this second definition to be a clinically significant PMI.

As mentioned earlier, renal injury was detected by early creatinine increase. If an increase in SCr >5% above baseline at 16 hours after PCI was detected, patients were defined as having significant ECS.

In addition, the association of baseline demographic, clinical, angiographic, and procedural characteristics with the occurrence of significant ECS was tested. Hypertension was defined as blood pressure >160/90 mm Hg on repeat measurements or current use of antihypertensive medications. Hyperlipidemia was defined as documented hyperlipidemia or use of lipid-lowering medications. Smoking status was defined as currently smoking or having quit within 6 months before PCI. Diabetes was defined as documented diabetes or use of insulin or oral hypoglycemic therapy.

### 2.1 Statistical analysis

The connection between ECS and the categorical variable parameters was examined with a chi-squared test. The association between ECS and the continuous variable parameters was analyzed using a simple logistic regression with ECS as a dependent variable. A multivariate logistic regression analysis was performed to determine variables independently connected with ECS. All variables that were associated with the outcome in the chi-squared test or the simple logistic regression analysis (at *P* = .1) were included in the multivariate regression. Statistical significance was considered at *P* < .05. Variables are presented as mean ± SD (in the case of continuous variables with normal data distribution) or as median ± IQR (in the case of continuous variables with nonnormal data distribution). All statistical analyses were performed using Statistica for Windows 12.0 software (Statsoft, Tulsa, OK).

## 3 Results

In this study, a total of 209 patients were enrolled, who completed all of the observations mandated by the protocol.

Baseline and procedural characteristics of the total study population are given in Table 1. The incidence of significant ECS in our study was 44.5% (93 patients). Characteristics of patients with significant ECS (group 1) and of patients without significant ECS (group 2) are shown in Table 2. Patients in group 1 were significantly more likely to have diabetes and had beta-blockers in their therapy less often. Other characteristics, including age, sex, obesity, medical therapy (except beta-blockers), history of hypertension, the presence of reduced eGFR, hyperlipidemia, PCI, and/or CABG, were similar in both groups.

**Table 1:**
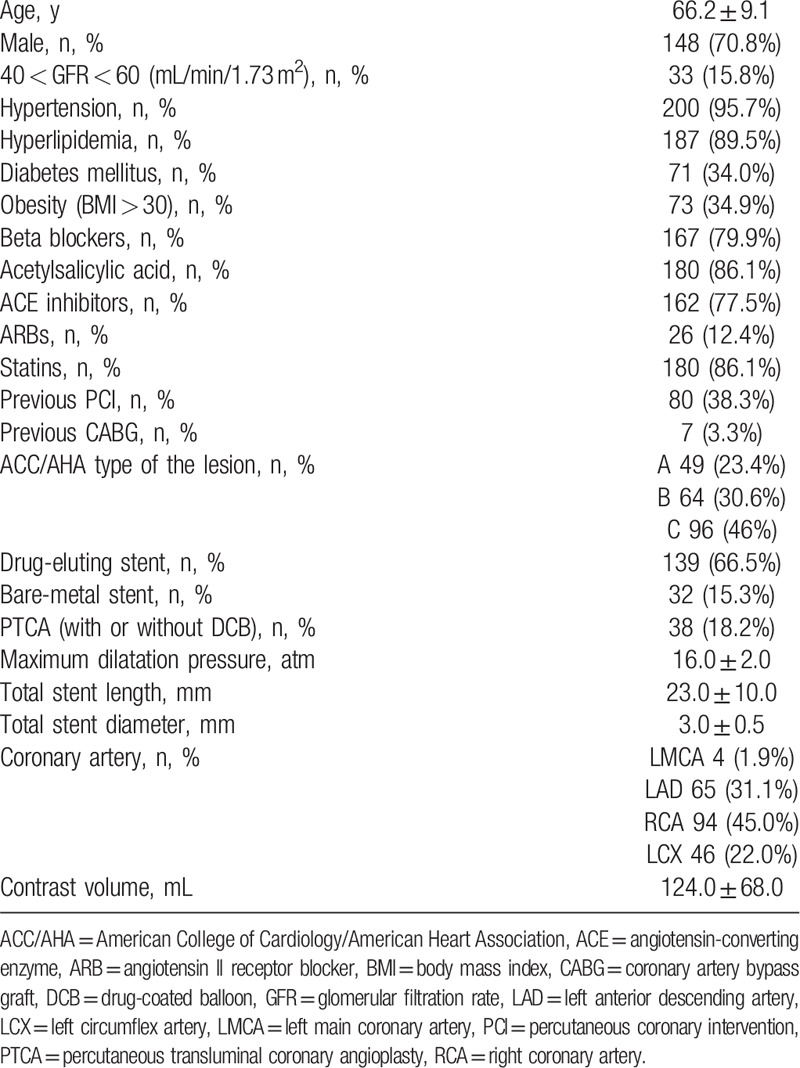
Baseline and procedural characteristics of the participants.

**Table 2:**
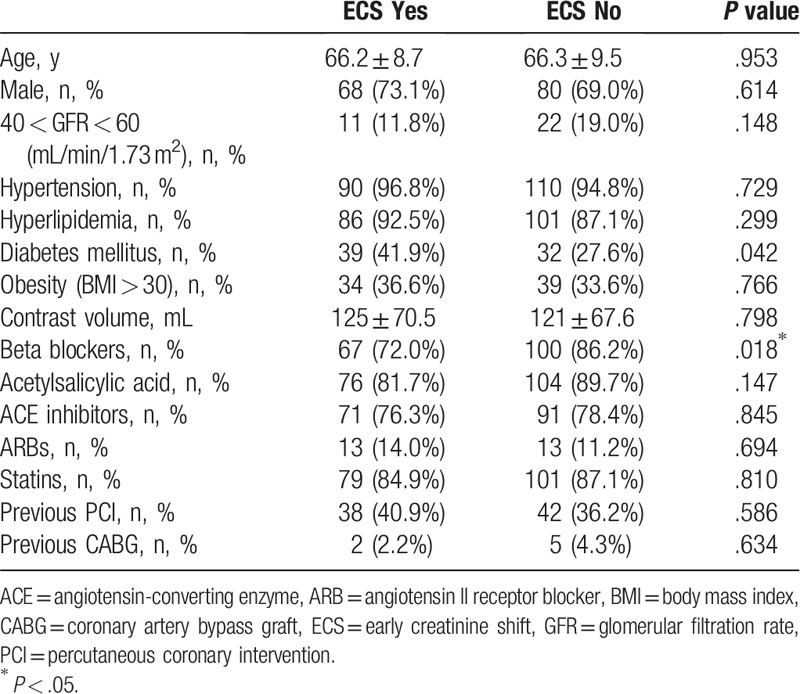
Characteristics of the participants and their correlation with ECS.

The overall incidence of PMI (defined according to the ESC consensus document) in our study was 77.5% (162 patients) among which 34.4% (72 patients) had low degree PMI, 43.1% (90 patients) had high degree PMI, and 29.7% (62 patients) developed clinically significant PMI.

We further tested the incidence and severity of PMI with respect to the incidence of significant ECS (Table 3). It was demonstrated that patients with PMI of a high degree had a significantly lower incidence of ECS (*P* = .007). Patients with low degree PMI and clinically significant PMI showed no significant association with ECS.

**Table 3:**
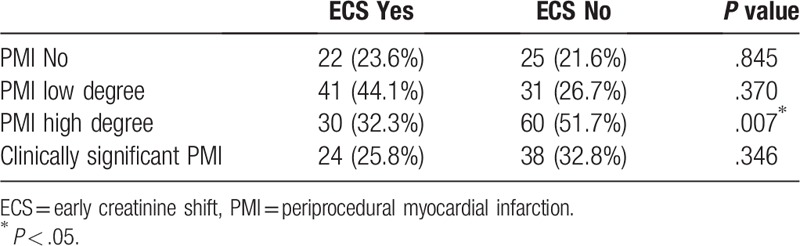
Association of PMI and ECS.

Table 4 shows multivariate analysis of the correlation of ECS with PMI of high degree and other factors that appeared to be significantly associated with ECS in the chi-square test or the simple logistic regression analysis (ie, history of diabetes mellitus, beta-blockers). Both parameters remained related to ECS in a statistically significant way in the multivariate analysis.

**Table 4:**
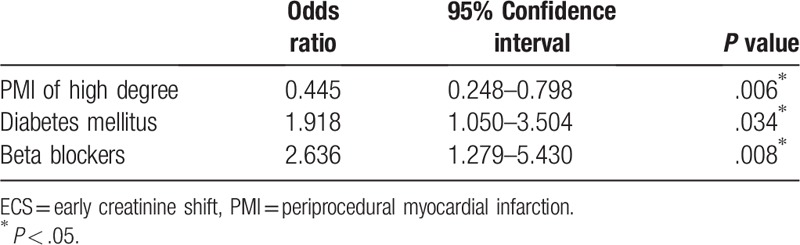
Multivariate analysis of association of PMI of higher degree, diabetes mellitus, and beta blockers in chronic therapy with development of ECS.

## 4 Discussion

Both PMI and CIN are serious and considerable complications of PCI associated with significantly worse outcomes. In our study, we aimed to assess whether there is any correlation between periprocedural myocardial and renal damage.

To execute our study, we overcame several challenges. The first problem was how to define PMI. We decided to incorporate both consensus documents as well as evidence-based data into our study. Furthermore, the universal definition of myocardial infarction was used as the consensus document. It defines PMI by a postprocedural rise of TnI in patients with or without basal elevation of preprocedural TnI. This definition further stratifies PMI into the low or high degree form.^[6]^

It is important to emphasize that this definition was based on conventional troponin assays. With the introduction of hsTn assays, which are able to detect even minimal concentrations of this biomarker, the uncertainty of the clinical value of hsTn increments following PCI has increased.^[19]^ Recently, studies have been performed to assess the incidence and clinical significance of hsTn elevations following uncomplicated elective PCI. The incidence of the postprocedural rise of hsTn above URL has been found to be around 60% to 80%, but the clinical significance of these findings remains controversial.^[18–20]^ Nevertheless, 2 large studies demonstrated a hsTn rise of ≥7x URL as the most accurate threshold to predict adverse cardiovascular events.^[17,18]^ Those 2 studies were the cornerstone of our evidence-based definition of PMI. They provided us both with the cut-off values for hsTnI as well as evidence of the clinical significance of those cut-offs. On the other hand, the consensus document relates to the conventional troponin, and it lacks data on the clinical significance of the proposed cut-offs once hsTnI is used. Taking all this into account, we decided to use a ≥7x URL rise of hsTnI as a cut-off for what we designed as a clinically significant PMI.

The next challenge in designing our study was the definition and detection of periprocedural renal injury. The most common definition of CIN today is an increase of 25% or more, or an absolute increase of 0.5 mg/dL or more, in SCr from the baseline value, 48 to72 hours following the exposure to CM.^[10]^ Given that the majority of patients currently undergoing invasive cardiovascular procedures are either outpatients or likely to be discharged within 24 hours after the procedure, the assessment of changes in SCr beyond 24 hours is often inconvenient.^[21]^ Recently, several studies have tested the importance of SCr increments in the first 24 hours after the procedure. The results showed that ECS even 12 hours after an elective PCI is a significant predictor of CIN and persistent renal damage.^[11,12]^ The results of those studies, as well as organizational issues inherent to our Department, lead us to use ECS at 16 hours as an early marker of CIN.

The main focus of our study was the association of PMI and ECS as a surrogate of CIN. Several author groups, including ours, have studied the association of PMI with renal impairment present at baseline. The studies have yielded conflicting results.^[22,23]^ However, we found no studies addressing the issue of concomitant renal and myocardial periprocedural injury. We tested different thresholds for PMI detection. These cut-offs were chosen according to the aforementioned principles, with the aim of finding one that would best correlate with renal injury. We found that PMI of a high degree, as was defined by the consensus document, was inversely associated with ECS. On the other hand, PMI of a low degree, as well as clinically significant PMI, showed no significant correlation with ECS. Although there were no previous studies regarding the connection of PMI and CIN, these results were quite the opposite of what we had expected. Given the significant overlap in both patient-related and procedure-related risk factors of PMI and CIN, we anticipated finding a positive correlation between these 2 conditions. Instead, we found an inverse relation or no relation. Although this phenomenon is difficult to explain, the fact that we used SCr values 16 hours after PCI instead of 48 hours, which is standard for CIN detection, could be the key factor for this unexpected association.

In 2002, a trial performed by Guitterez et al, which included 98 patients undergoing cardiac catheterization, found that the measurement of SCr within 24 hours was not helpful in anticipating SCr trajectory.^[24]^ On the other hand, a clinical trial performed by Ribichini et al, which included 216 at-risk patients undergoing CA, demonstrated that a 5% increase in SCr from the baseline at 12 hours was a sensitive (75%) and specific (72%) marker of CIN at 48 hours and the persistent worsening of renal function at 30 days.^[11]^

Another trial performed afterward by Ribichini et al, which included 166 at-risk patients undergoing CA and interventions, showed that a 5% increase in SCr from the baseline at 12 hours was a sensitive (70%) and specific (76%) marker of CIN at 48 hours.^[12]^ In both of these studies, the incidence of CIN was 18%. However, neither of the 2 Ribichini et al studies reported the incidence of ECS. Instead, they mentioned that early SCr increase is a useful prognostic marker for CIN with significant sensitivity and specificity.

Since most of our patients were discharged within 24 hours after PCI, we also assessed ECS as a marker of CIN development. In our study, the incidence of ECS was around 45%, which was far more than we had anticipated, especially considering that the majority of our patients had mild chronic kidney disease with eGFR ≥60 mL/minute. Unfortunately, previous studies did not provide us with exact information about the incidence of ECS. Nevertheless, judging by the incidence of CIN in studies performed by Ribichini et al, as well as their calculations of specificity and sensitivity of early SCr increments in predicting CIN, we estimated that the incidence of ECS in their research was substantially lower than in ours. The exact reasons for such discordance are not entirely clear. While there were some interstudy differences, no major distinctions in the number of patients and their characteristics were found. However, we have to emphasize the possible selection bias in our study, as we only included patients with eGFR ≥40 mL/minute, and measurement bias considering the fact that in our study blood samples were tested 16 hours after the procedure as opposed to 12 hours in Ribichini et al trial. Furthermore, in Ribichini et al trial, all patients were intravenously hydrated before the procedure, while in our research they were not, as current guidelines do not strictly recommend preventive intravenous hydration in patients with eGFR ≥40 mL/minute.^[2,9]^ It is important to emphasize that patients with eGFR < 40 mL/minute were excluded from our study. We decided on such an approach because different hydration protocols were used for such patients, and we felt that it could seriously influence our results. Such a decision could also be responsible for the differences in the results. Although the 4-hour difference in taking blood samples, patient selection, and intravenous hydration could give us some clues, it is likely that there are other causes responsible for our notably divergent results. Unfortunately, there are no studies addressing that issue. Therefore, given the small sample size of the current studies as well as their different outcomes, the prognostic significance of early changes in SCr remains, at the very least, debatable. Nevertheless, it is worth studying the interesting potential of the percentage change of SCr within 24 hours to be a simple, easily accessible and low-cost parameter to provide early identification of patients with a high risk of CIN. Future studies comprised of a larger number of patients are needed to determine the most accurate threshold for early CIN detection or to rule out its potential benefit.

By performing further statistical analysis, we attempted to find other clinical factors that could (possibly) be associated with ECS. According to previous studies, diabetes and chronic kidney disease are well-known risk factors for CIN.^[13,14]^ Together with the administration of iodinated radiocontrast agents, diabetes is associated with renal impairment through different pathophysiological mechanisms as shown by several authors.^[26]^ In addition, patients with already impaired renal function are even more sensitive to the deleterious impact of CM.^[10]^

In our study, we found no association between reduced basal eGFR and ECS. However, patients with diabetes had a notably higher incidence of early SCr increments. This finding aligns with the results of previous trials regarding diabetes and CIN association. Hence, it indicates that the concept of ECS deserves further investigation.

Furthermore, beta-blockers demonstrated a marked inverse association with ECS while other medications showed no significant correlation. Previous studies did not reveal a significant reduction in CIN incidence with the use of beta-blockers.^[27,28]^ Given the relatively small number of patients in our study and the possible pitfalls of ECS as a surrogate of CIN use, the potential benefit of beta-blockers should be tested in additional trials with a different design. In conclusion, our results are at least a little surprising.

We expected to find some association of renal and myocardial periprocedural damage. Surprisingly, our results show that there is either no association or even some inverse relation of those 2 clinical phenomena. The most probable explanation lies either in the imperfection of the way in which PMI was defined or in the questionable concept of ECS as a marker of CIN. We attempted to mitigate concerns about the former by using both definitions of PMI proposed by relevant cardiologic associations as well as definitions that are proven to predict adverse clinical events. The clinical implications of ECS as an early marker of CIN, especially in the era of the SDD PCI, seems extremely attractive.^[29]^ Unfortunately, it has not been tested by many author groups. Although especially when analyzing our results, its accuracy may be questioned, we strongly believe that this concept, due to its practical nature, should be further investigated. The results of our study could be considered as a test of the ECS concept. To fully investigate the association of PMI and CIN, one should have a clear-cut definition of PMI as well as a 48 to 72 hour patient observation period in order to track any SCr level changes. If an association would be found, PMI as an early predictor of CIN could provide significant clinical implications in the era of the SDD PCI. Clearly, further investigations should be done, and our article points to the need for such further studies.

## 5 Limitations

The current report is a cross-sectional analysis, and, therefore, the results and conclusions are subject to the limitations inherent in all such reports. In addition, we had a relatively small sample size. Other biases such as the PMI definition used as well as the ECS concept are extensively elaborated in Section 4.

## 6 Conclusion

PMI and CIN are complications of PCI that are relatively frequent and are both associated with adverse clinical events. Similar risk factors for both phenomena make their association highly likely. Yet, this possible association was not extensively studied to date. In our study, we did not find a positive correlation, as we initially expected, between PMI and a surrogate of CIN, that is, ECS. Future studies with larger number of participants and longer patient observation period are needed to determine a possible association of PMI and CIN. Such studies could serve to further investigate the attractive clinical implications of both ECS as a surrogate of CIN and PMI as a potential predictor of CIN.

## Acknowledgments

The authors thank Mr Grgur Valentic, master's degree in mathematics, for the assistance in carrying out the statistical analysis in this paper.

## Author contributions

**Conceptualization:** Tomislav Letilovic.

**Formal analysis:** Ivana Jurin, Vedran Radonic.

**Investigation:** Mario Stipinovic, Luka Percin, Ivana Jurin.

**Methodology:** Helena Jerkic, Vedran Radonic.

**Project administration:** Ivana Jurin.

**Software:** Vedran Radonic.

**Supervision:** Tomislav Letilovic.

**Validation:** Helena Jerkic.

**Visualization:** Mario Stipinovic.

**Writing – original draft:** Mario Stipinovic, Luka Percin.

**Writing – review & editing:** Tomislav Letilovic.
